# Bibliometric Keyword Analysis across Seventeen Years (2000–2016) of *Intelligence* Articles

**DOI:** 10.3390/jintelligence6040046

**Published:** 2018-10-15

**Authors:** Bryan Pesta, John Fuerst, Emil O. W. Kirkegaard

**Affiliations:** 1 Department of Management, Cleveland State University, Cleveland, OH 44115, USA; 2 The Ulster Institute for Social Research, London AL2 1AB, UK; j122177@hotmail.com (J.F.); the.dfx@gmail.com (E.O.W.K.)

**Keywords:** bibliometrics, *Intelligence*, keywords

## Abstract

An article’s keywords are distinct because they represent what authors feel are the most important words in their papers. Combined, they can even shed light on which research topics in a field are popular (or less so). Here we conducted bibliometric keyword analyses of articles published in the journal, *Intelligence* (2000–2016). The article set comprised 916 keyword-containing papers. First, we analyzed frequencies to determine which keywords were most/least popular. Second, we analyzed Web of Science (WOS) citation counts for the articles listing each keyword and we ran regression analyses to examine the effect of keyword categories on citation counts. Third, we looked at how citation counts varied across time. For the frequency analysis, “g factor”, “psychometrics/statistics”, and “education” emerged as the keywords with the highest counts. Conversely, the WOS citation analysis showed that papers with the keywords “spatial ability”, “factor analysis”, and “executive function” had the highest mean citation values. We offer tentative explanations for the discrepant results across frequencies and citations. The analysis across time revealed several keywords that increased (or decreased) in frequency over 17 years. We end by discussing how bibliometric keyword analysis can detect research trends in the field, both now and in the past.

## 1. Introduction

Bibliometrics is the branch of library science that applies mathematical and statistical techniques to analyze books, articles, and other documents [1,2]. The discipline is becoming increasingly popular. For bibliometric evidence of this claim, note that the correlation between the number of published articles on “bibliometrics” (via Google Scholar), and the year of publication (this century, through 2017) is positive and very strong (0.91).

Bibliometric techniques are also convenient, as the relevant data are both quantitative and readily available [3]. Moreover, the field has high utility. Bibliometrics can be used to inform policy decisions [4], allocate research funding [5], assist libraries in prioritizing acquisitions [6], and of course, evaluate scholarly activity [7]. Even a “bibliometric study of literature on bibliometrics” has now been conducted [8]. The field is therefore likely here to stay, and will continue to be a primary means of judging impact for articles, researchers, and journals.

The present article focuses on bibliometrics for the journal, *Intelligence*. The journal was founded in 1977 by Douglas K. Detterman. Since then, it has published 1828 articles [9], including two that feature bibliometric analyses. First, Wicherts [10] analyzed Web of Science (WOS) citation counts for 797 articles in the journal, published between the years 1977 and 2007. The median citation count for these articles was 10, with a mode of 6. Wicherts also showed that the journal’s impact factor had been steadily rising each year, although it has since leveled off.^1^ Finally, Wicherts reported a top 25 list of the most-cited papers in the journal to date. These articles had citation counts (via Google Scholar) ranging from 81 to 492.

Second, Pesta [11] examined articles published after Wicherts’ study. He analyzed 619 papers, written by 1897 authors, and published between the years 2008 and 2015. Pesta reported citation counts for both articles and authors, and found the median citation rate for the former (i.e., 10) to be identical to that calculated by Wicherts (albeit, Pesta’s mean citation value was 17, with a mode of 6). Pesta also reported a list of the most prolific authors, and an updating of the 25 top, most-cited articles in the journal between the years 1977 and 2015. These articles had citation counts (via Google Scholar) ranging from 186 to 905.

Here we attempt to build upon existing bibliometric research for this journal. Our focus, however, is on the article keywords, versus the authors, or the articles themselves. Note that the journal began using keywords in the year 2000.

Why apply bibliometrics to article keywords? First, keywords represent the author’s opinion of the three to five (or so) most important words in their articles. Second, keyword analysis can potentially detect trending research topics both currently, and in the past. Third, bibliometric keyword analysis can answer several interesting questions. Some of these include (1) What research topics in this journal are the most frequent/popular; (2) Are certain keywords associated with an increased likelihood of a paper being cited; and (3) Has the use of specific keywords increased or decreased over time? Our goal is to provide bibliometric answers to these questions, focusing specifically on articles and keywords published in the journal, *Intelligence*.

## 2. Method

We coded all keyword-containing articles in the journal, *Intelligence*, for the years 2000 to 2016. In line with other bibliometric studies on this journal [10,11], we included all articles except book reviews and obituaries. Note that the year 2000 was the first we found where the journal featured keywords, although only one such article appeared at this time (i.e., [12]). We did not code post 2016 articles because we did not think they had enough time to accumulate a relative, representative number of citations. One of the prior bibliometric reviews of *Intelligence* [11] did similarly. Moreover, we excluded some other articles published after 2000 (e.g., book reviews, obituaries, and some editorials), because they did not contain keywords. In total, we ended up coding 916, keyword-containing articles.

For each article, we coded the title, first author’s name, and all listed keywords. Regarding keywords, we coded 4364 of them from the articles in the set. On 11 July 2018, we next coded each article’s Web of Science (WOS) citation count. We did not also code Google Scholar (GS) counts, as Pesta [11] reported that these correlate 0.97 with WOS citation counts. In fact, GS counts are very close to double those reported by WOS. Note that these values come specifically from analyzing articles published in *Intelligence* [11].

We coded both citations overall and citations per year for every keyword in the set. The latter values adjust citation counts for the effects of “time since publication” on a paper’s current citation count [11]. In line with a previous review [11], this calculation involved dividing each article’s number of citations by (2018.53 minus the article’s year). We used 0.53 as the decimal because 11 July is the 192nd day of the year, and 192 divided by 365 equals 0.53.

Coding the keywords was sometimes not straightforward. For example, authors often used several different keywords to describe the same research topic (e.g., “g”, “g factor”, “general mental ability”, “general cognitive ability”, and “general intelligence”). This required us to form dozens of “keyword categories”, each containing all synonym keywords for the same underlying construct. An example of a keyword category would be “g factor” for the synonyms listed in parenthesis above.

Next, within articles, authors often used keywords that were redundant (e.g., “general intelligence” and “g”). In fact, redundant keywords comprised 763 of the 4364 (17.5%) keywords in the article set. However, counting all redundant keywords within articles would artificially inflate (i.e., double count) overall citation rates for both the articles and the keywords. We therefore did not analyze redundant keywords. Finally, a small number of keywords could logically be placed into more than one category (e.g., “speeded and un-speeded testing”). Although only eleven (2.50%) such keywords existed, we nonetheless excluded them from analyses.

The first two authors separately coded all keywords into categories. We then compared the codings to identify discrepancies. These were discussed until consensus was reached on every keyword category. Most discrepancies involved disagreements on how fine to delineate categories (e.g., whether to group “general intelligence” separate from “intelligence”). As such we did not calculate a reliability coefficient, since discordance often reflected a difference in level of analysis, not an inconsistency in classification.

Unexpectedly, judging from our preliminary review of the first couple of years of data, many of the resulting keyword categories turned out to have very small sample sizes (i.e., number of articles listing them). We therefore chose to analyze only keyword categories with at least 20 citations. There were 38 such unique keyword categories (37 after excluding “intelligence”), with 2699 (2161 after excluding “intelligence”) references to these keywords in the article set.

We ran three separate analyses on the keyword categories. The first was a simple frequency comparison of how many articles listed each keyword. This allowed us to identify the most frequent/popular research topics. The second involved mean difference tests of WOS citation counts for the articles citing each of the 37 keyword categories, together with regression analyses of the effect of keyword categories on citation counts. The goal here was to determine which categories were associated with highly cited papers.

Third, for the categories associated with the most paper citations (i.e., those categories with at least 50 paper citations; *N* = 20, which is 54% of the 37 keyword categories), we visualized WOS trends in citation counts across time. Specifically, we created plots with year on the x-axis and proportion on the y-axis. The proportion was simply the number of articles citing the keyword in a given year, divided by the total number of citing articles for that keyword. This approach allowed us to visually spot trends in keyword citations over the years.

## 3. Results

Table 1 lists frequency data for the 37 keyword categories (hereafter, “keywords”) that we analyzed. The third column shows how many times the keyword was listed by one of the 916 articles in the set. Also reported are standardized residuals. These can be interpreted as *Z* scores [13,14]. Residuals of greater than plus or minus two indicate that the keyword’s observed frequency was significantly higher (or lower) than its expected frequency.

Not surprisingly, “intelligence” was the most frequently employed keyword. It was listed as a keyword by 59% of the articles in the set. Because “intelligence” is the primary focus of the journal, we decided not to include it in the analyses that follow.

Next, by a fair amount, the keyword associated with the most number of citations (excluding “intelligence”) was “g factor”. It was listed by 15% of the articles in the set. This is also not surprising, but it perhaps exemplifies the field’s sustained, direct focus on general intelligence, versus specific cognitive abilities. For example, only 7 of the 916 (0.01%) articles in the set included “non-g abilities” as a keyword (but see also the frequencies for crystallized and fluid intelligence in Table 1).

“Psychometrics/statistics” was the second-most listed keyword in the set. This seems intuitive, as these are the tools researchers must use to get their data published in the journal. Next was the keyword, “education”. It ranked third on the list, which seemed surprising, given our perhaps outdated impression that educational researchers tend to eschew intelligence research. On the other hand, at least one seminal article on this topic appears in *Intelligence* [15]. The article is the third-most cited paper of all time for the journal. Moreover, “education” might attract relatively more research interest because the keyword is broadly multi-disciplinary. The supply of researchers able and interested in this topic may be greater than that for many of the other keywords in the article set. This explanation is admittedly speculative.

The fourth most-listed keyword was “IQ/achievement/aptitude tests”. This keyword is a hodgepodge, which likely explains its relatively high count. For example, the category contains 102 keyword synonyms total. Of these, 84 (82.4%) are unique (i.e., were listed by only one article in the entire set). Some of the many exemplars for this category include “AFQT”, “CAT”, “Draw a Person”, “GATB”, “GMAT scores”, “Stanford Binet”, “TIMSS”, “WAIS III”, “WISC”, and “Wonderlic”. Rounding out the top-five listed keywords in Table 1 was “Race/ethnicity”. We attribute this keyword’s high frequency count partly to the work of Richard Lynn and colleagues; see, e.g., [16], the authors of which have mapped out IQs for numerous ethnicities/national origins across the world.

Finally, we are reluctant to discuss the keywords in Table 1 that have relatively low frequencies. These are misleading. Keywords like “emotional intelligence”, “politics”, and “Spearman’s hypothesis” have “low” citation counts, but only relative to the other keywords in Table 1. Recall that Table 1 presents just the top 37 out of 384 (9.6%) categories. Therefore, any keyword appearing in Table 1 is indeed something multiple researchers have expressed interest in. Conversely, the keywords with relatively lower impact would be those that did not make Table 1.

Table 2 shows WOS citation rates for the top 37 keywords in the set. Specifically, for every article listing a keyword, we coded that article’s WOS citation count, and then took the average of all article counts within each keyword. We report both citation counts overall, and then per year.

Interestingly, the top 5 keywords in Table 2 are all different from those in Table 1. Several of the keywords were associated with more citations, relative to keywords with higher frequencies of usage. For example, “spatial ability” ranked first (55.53 WOS cites) in Table 2, but only 29.5th (32 counts) in Table 1. Conversely, “psychometrics/statistics” ranked second in Table 1, but only 15th in Table 2. This led us to correlate the ranks across frequencies (Table 1) and overall citations (Table 2). Although the resulting correlation was actually negative (*r* = −0.192), it was small and not significant (*p* = 0.369). Still, the safest interpretation is perhaps interesting: publishing on a frequently-researched keyword does not guarantee that the article will yield the highest citation counts.

In Table 2, the keyword associated with the second highest number of citations was “factor analysis”. We see this as paralleling “psychometrics/statistics” in Table 1. Specifically, both keywords represent tools that researchers use when attempting to publish in this journal. Third, “executive function” is another example of a relatively infrequently used keyword associated with a high number of citations. However, the construct is very similar to “working memory” (we almost grouped these two keywords together—see, e.g., [17]), which is a staple research topic in this journal. It has both a correspondingly high frequency and associated citation count (i.e., it ranked sixth of all keywords in both Table 1 and Table 2).

“Attention” was the keyword associated with the fourth highest number of citations. This is another illustration of a keyword with a relatively low frequency (25th in the rank) but a high number of citations. Moreover, the keyword has seven articles referencing it, each with at least 50 citations. Two of these [18,19] even fall on Pesta’s [11] top 25 list of all time, with 224, and 479 overall citations, respectively. Finally, “IQ theories” was the keyword associated with the fifth highest number of citations. This keyword is also a hodgepodge (made up of, e.g., “multiple intelligences”, “VPR theory”, “practical intelligence”, and “Gf–Gc theory”), but we have no real explanation for why papers using these keywords amassed such high citation counts.

The top-five values for citations per year in Table 2 are mostly similar to the values for citations overall in Table 1. An exception is that “working memory” replaced “IQ theories” in cites per year versus cites overall. Also noteworthy is that the correlation between the two citation values for all keywords in Table 2 is 0.91. In sum, no very large differences in ranks occurred when looking at citations overall versus per year.

Turning to statistical analyses, the Table 2 grand mean for citations overall (*N* = 2161) was 26.28 (*SD* = 42.53). A one-way ANOVA on these data was significant: *F* (36) = 2.49; *MSe* = 1765. Similarly, the grand mean for citations per year was 2.89 (*SD* = 3.51). This ANOVA was also significant: *F* (36) = 3.30; *MSe* = 11.90. For context, recall that both Wicherts [10] and Pesta [11] reported median citation counts of 10 for all articles in their sets. The keywords in Table 2 (i.e., the top 37 in the entire journal) range from being cited 2.63 times to substantially more than that (e.g., papers listing “spatial ability” as a keyword averaged 55.53 citations each).

The one-way ANOVAs above each have 37 levels. This creates an awkward scenario when trying to determine how best to run post-hoc tests. Ultimately, we decided not to conduct Family Wise Error Rate tests, as the number of multiple comparisons here was too large: (37 × 36)/2 = 666. A correction like Bonferroni’s would have an unduly punitive effect on our statistical power. Specifically, the effective alpha rate with a Bonferroni correction on these data would be 0.05/666 (0.00008).

Instead, we employed the false discovery rate (FDR), which is particularly useful when researchers conduct many post hoc tests. The FDR is the proportion of Type I errors existing among all tests that resulted in rejecting the null hypothesis. This is in contrast to the typical reliance on the alpha level (i.e., *p*-value) to determine Type I error rates. Instead, the FDR focuses on the *q*-value (i.e., the proportion of significant comparisons that are actually Type I errors). A *q*-value of 0.05 means that 5% of the significant test results are likely Type I errors [20,21].

The Benjamini–Hochberg test [21,22] is a common procedure used to control for the FDR. It does so via calculation and interpretation of *q*-values. We used it here for our post hoc tests. Following convention, we adopted 0.05 as our value for *q*.

Fully 70 of the 666 (11%) post-hoc comparisons for overall citations were significant with *q*-values of less than 0.05. The results for citations per year were similar, yielding 105 of 666 (16%) significant comparisons. The Supplementary Materials lists all pairwise comparisons, both for citations overall, and then per year.

We next analyzed the effects of keywords on citation counts by running a regression model with keyword, publication year (and nonlinear transformations of this), and keyword count as independent variables. The dependent variable was the citation number. A reviewer suggested that we add a dummy variable for the editor to capture the effect of editorial preference; however, Doug Detterman was editor-in-chief from 2000 to 2016, when Richard Haier took over. As our data extends to 2016, there is little to no editorial variance (we also do not know who the action editor was for each paper). Results appear in Table 3. Because the analysis involves the full population of *Intelligence* papers (versus some sample of them), statistical significance is arguably not an important concern. Nonetheless, we asterisked those keywords that had *p*-values <0.05, and then used these as a threshold to warrant further discussion. Results from other models are shown in the Supplementary Materials. The model we selected here was geared toward finding the highest *R*^2^-adj-value.

Results below parallel those observable from the FDR analyses. Papers dealing with “executive function”, “factor analysis”, “fluid intelligence”, “IQ theories”, “working memory”, and “spatial ability” were all cited as more than typical, while those dealing with “health”, “mental speed”, and “race/ethnicity” were cited as less than typical. It is no secret that “race/ethnicity” is an unattractive topic for many researchers, so it is perhaps not surprising that papers focusing here garner lower citations. The topic of cognitive epidemiology (“health”) may be similarly less popular. Finally, “mental speed” and “ECTs” were often conceptually overlapping categories, and both of their betas were negative. The reason for the negative effects of these topics is unclear.

Our final analysis involved looking at trends in citation counts over the years. That is, does a keyword’s popularity (operationalized as the mean citation count of the articles that listed it) change over time? A preliminary way of testing this is to simply correlate the year an instance of a keyword appeared with its corresponding WOS citation counts.

This correlational analysis included every exemplar (*N* = 2161; “intelligence” excluded) of the top 37 keywords in Table 2. The year of publication correlated moderately at −0.375 with overall WOS citations. However, in looking at the scatterplot (not displayed here), the inverse relationship occurred because many of the papers with very high citation counts were published prior to 2010. Consistent with this, the correlation between year of publication and citations per year (which corrects for time since publication) was only −0.148. This value, though, was still significant, given the large sample size.

**Figure 1:**
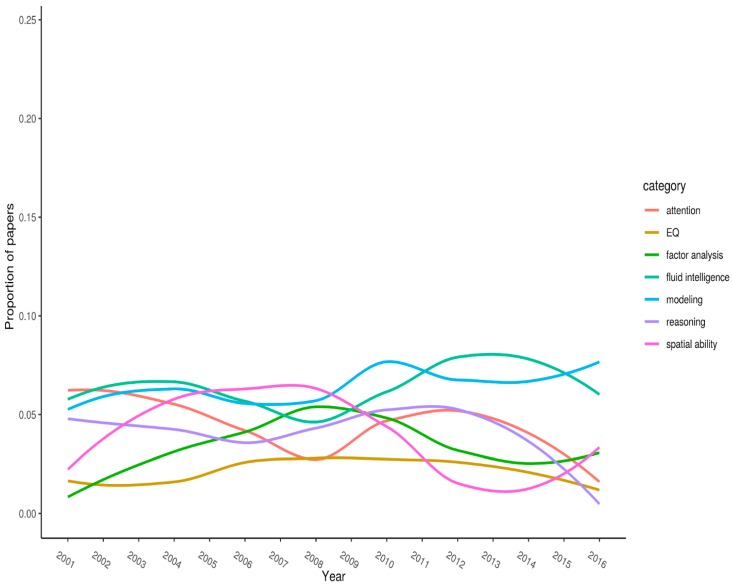
Proportion of articles each year that list a given keyword (flat).

**Figure 2:**
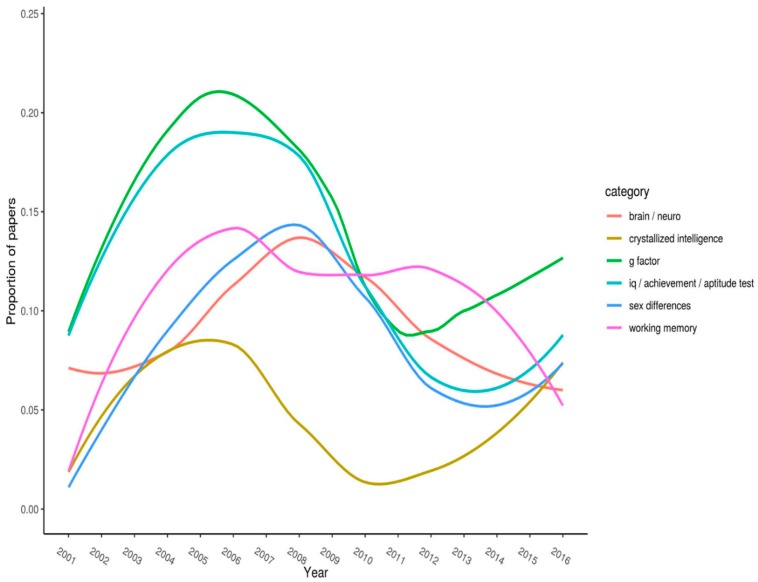
Proportion of articles each year that list a given keyword (parabolic).

**Figure 3:**
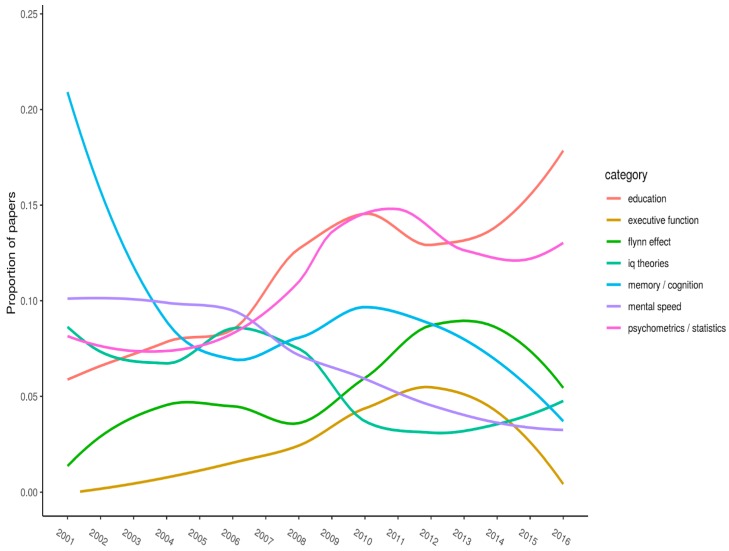
Proportion of articles each year that list a given keyword (increasing/decreasing).

A more revealing analysis involves plotting the change in citations for specific keywords across the years. We deemed that visual presentation of these data would be the easiest way to interpret them. Figure 1, Figure 2 and Figure 3 (split into three panels illustrating flat, parabolic, and increasing/decreasing trends, respectively) shows the top-20 keywords. Each keyword has *n* > 50 citations overall. The year of publication is plotted on the x-axis, and the proportion of articles (published in the journal that year) with the specified keyword is plotted on the y-axis.

Note the timespan for where curves peak on the y-axis. Peaks represent when keywords received (proportionately, compared with all other articles published that year) their most amount of research attention. For example, “g-factor” peaked between 2004 and 2008, after which it steadily declined (it has, however, recovered somewhat over the last few of years).

Figure 1 displays keywords that appear to show little trend. These tend to have relatively low frequencies. This set includes “attention”, “EQ”, “factor analysis”, “fluid intelligence”, “modeling”, “reasoning”, “and spatial ability”.

Figure 2 shows keywords that appear to exhibit a marked nonlinear relation. Notably, between about 2004 and 2008, “g factor” and “IQ/achievement/ability tests” peaked. Likewise, around years 2007–2010, “brain/neuro” and “sex differences” hit their peaks. They thereafter trended downward. Finally, “crystallized intelligence” showed a strong drop off between 2009 and 2011 with a recovery over the last five years.

Figure 3 shows keywords that exhibit somewhat linear trends. “Education” is currently showing a strong trend upward, as is the “Flynn effect”, except for in the most recent years. Conversely, “memory and cognition” is showing a downward trend, as is “mental speed” and possibly “IQ theories”. Additionally, “psychometrics/statistics” has somewhat increased. This is a hodgepodge category, so little can be made of this trend. We included “executive function” in Figure 3 as well, since it was increasing until just recently.

## 4. Discussion

For several reasons, we decided to conduct bibliometric analyses on keywords for articles (2000–2016) published in the journal *Intelligence*. First, we were interested in which keywords were most often employed by authors publishing articles in this journal. Next, we wondered if certain keywords were associated with greater or fewer citations for the papers that listed them. Lastly, we sought to identify trending keywords—ones that had increased or decreased in usage over the 17-year span where the journal started featuring them.

Summarizing, the five-most frequently listed keywords (Table 1; “intelligence” excluded) were “g factor”, “psychometrics/statistics”, “education”, “IQ/achievement/aptitude tests”, and “race/ethnicity”. These keywords accounted for 574 of the 2161 (27%) total keyword instances in Table 1.

Regarding WOS citations for articles by keywords, those with the highest means overall were “spatial ability”, “factor analysis”, “executive function”, “attention”, and “IQ theories”. Together, articles using these keywords averaged 47.1 citations overall. This is in contrast to a median citation rate of ten (for all articles in the journal), reported by both Wicherts [10], and Pesta [11]. However, we found it counter intuitive that the top-five most frequently listed keywords were all different from the top-five keywords with the highest mean citation values. We tentatively conclude there is a low correlation between a keyword’s frequency of use, and how many citations it will receive on average from the papers that list it.

But what could explain the discrepancy? A reviewer suggested a plausible scenario: An article using a low frequency keyword will tend to be one of only very few addressing the respective research question. Therefore, if others conduct research in this area, it is more likely that they will cite the article in their own work. Thus, this article might be cited relatively frequently despite the keyword being used infrequently overall. In contrast, an article using a very frequent keyword will probably be only one among many others that could be cited. Therefore, the article might have a relatively lower probability of being cited by newer papers in the area.

Our last analyses was an attempt to identify trends across time for the most frequently listed keywords in the article set. We visually displayed these trends in Figure 1, Figure 2 and Figure 3. As might be expected, no keyword’s frequency increased (or decreased) monotonically across the years. Instead, all trends were curvilinear. Examples of keywords with notable increases in frequency across recent years included “crystallized intelligence” and “education”. Those with notable decreases were “brain/neuro” and “executive function”.

Regarding study limitations, perhaps the largest is that we had no other, similar journal’s keyword data to serve as a reference point or control group. We did, however, compare our findings with those in [10,11] where appropriate. Next, coding the keywords was not completely objective. Many authors used synonym keywords across articles, and/or redundant keywords within articles.^2^ We attenuated this problem by having multiple raters rank and discuss each of the codings.

Nonetheless, we were forced to use keyword categories, versus each specific keyword itself, due to the high frequency of synonym keywords across papers. Perhaps the journal should implement a more standardized approach to author keyword selection. One way this could be achieved is for the journal to present a drop down list of keywords that authors select from when submitting their manuscripts. This approach could provide benefits beyond just the facilitation of bibliometric analyses. For example, standardizing keywords would help readers more efficiently find articles they are interested in.

## 5. Conclusions

In sum, the present paper was a first step toward illuminating how keyword analysis can shed light on this journal’s focus. Specifically, we reported which research topics authors spent the most time on, and of them, which averaged the most citations. We also identified keywords that trended across the 17-year time span of our article set. It is our hope that periodic bibliometric analyses of publications in this journal will continue to generate big picture data on where the field has been, and where it might be going.

## Figures and Tables

**Table 1:** Frequencies, percentages, and residuals for the 37 keywords with the most counts in the article set.

Rank	Keyword	Frequency (%)	Residual	Standardized Residual
-	Intelligence/cognitive ability	538 (58.7%)	-	-
1	g factor (general intelligence factor)	141 (15.4%)	82.6	10.81
2	Psychometrics/statistics	116 (12.7%)	57.6	7.54
3	Education	114 (12.5%)	55.6	7.28
4	IQ/achievement/aptitude tests	102 (11.1%)	43.6	5.71
5	Race/ethnicity	101 (11.0%)	42.6	5.57
6	Working memory	97 (10.6%)	38.6	5.05
7	Brain/neuro	84 (9.2%)	25.6	3.35
8	Nature/nurture	81 (8.8%)	22.6	2.96
9	Children/child development	76 (8.3%)	17.6	2.30
10	Memory/cognition	74 (8.1%)	15.6	2.04
11.5	Sex differences	73 (8.0%)	14.6	1.91
11.5	Income/status/SES	73 (8.0%)	14.6	1.91
13	Health	70 (7.6%)	11.6	1.52
14	Adult/aging	69 (7.5%)	10.6	1.39
15	Flynn effect	61 (6.7%)	2.6	0.34
16	Fluid intelligence	60 (6.6%)	1.6	0.21
17	Modeling	58 (6.3%)	−0.4	−0.05
18.5	ECTs (Elementary cognitive tasks)	57 (6.2%)	−1.4	−0.18
18.5	Genes/evolution	57 (6.2%)	−1.4	−0.18
20	Mental speed	51 (5.6%)	−7.4	−0.97
21	IQ theories	49 (5.4%)	−9.4	−1.23
22	Aggregate/regional IQs	47 (5.1%)	−11.4	−1.49
23	Raven’s	45 (4.9%)	−13.4	−1.75
24	Crystallized intelligence	39 (4.3%)	−19.4	−2.54
25	Attention	36 (3.9%)	−22.4	−2.93
26	Personality	34 (3.7%)	−24.4	−3.19
27.5	Reasoning	33 (3.6%)	−25.4	−3.32
27.5	Executive function	33 (3.6%)	−25.4	−3.32
29.5	Factor analysis	32 (3.5%)	−26.4	−3.45
29.5	Spatial ability	32 (3.5%)	−26.4	−3.45
31	Spearman’s Hypothesis	31 (3.4%)	−27.4	−3.59
32	Item level/IRT (Item response theory)	28 (3.1%)	−30.4	−3.98
33	Politics	23 (2.5%)	−35.4	−4.63
34	Longitudinal designs	22 (2.4%)	−36.4	−4.76
35.5	SLODR (Spearman’s law of diminishing returns)	21 (2.3%)	−37.4	−4.89
35.5	Problem solving/decision making	21 (2.3%)	−37.4	−4.89
37	EIQ (Emotional intelligence)	20 (2.2%)	−38.4	−5.02

Notes: The frequencies are out of 916 articles and 2699 (2161 without “intelligence”) keyword counts. The resulting expected value for each cell is 58.4. Lastly, “intelligence/cognitive ability” was not included in statistical analyses for Table 1.

**Table 2:** Mean Web of Science (WOS) citation counts for articles with specific keywords.

WOS Rank	Keyword	WOS Cites *M* (*SD*)	WOS Cites Per Year *M* (*SD*)
-	Intelligence/cognitive ability	24.89 (43.93)	2.82 (3.80)
1	Spatial ability	55.53 (71.38)	4.67 (4.88)
2	Factor analysis	54.50 (98.73)	4.98 (7.42)
3	Executive function	42.33 (49.17)	5.80 (6.03)
4	Attention	42.08 (84.58)	3.96 (5.28)
5	IQ theories	40.96 (56.23)	3.93 (5.24)
6	Working memory	39.87 (61.06)	4.39 (4.72)
7	Memory/cognition	31.46 (63.89)	2.94 (3.98)
8	Fluid intelligence	30.82 (39.65)	3.86 (4.12)
9	IQ/achievement/aptitude tests	29.35 (59.20)	2.88 (5.11)
10	Modeling	28.59 (63.27)	3.24 (4.21)
11	Sex differences	28.49 (28.70)	2.89 (2.31)
12	Education	28.39 (58.00)	3.31 (5.12)
13	g factor (General intelligence factor)	27.25 (32.14)	2.71 (2.49)
14	Reasoning	26.24 (35.70)	2.92 (3.21)
15	Psychometrics/statistics	26.05 (41.35)	2.84 (3.37)
16	Crystallized intelligence	25.62 (37.29)	2.73 (3.25)
17	Flynn effect	25.41 (30.18)	3.14 (2.28)
18	Mental speed	25.24 (30.31)	2.44 (2.24)
19	EIQ (Emotional intelligence)	24.45 (23.68)	2.88 (1.98)
20	Brain/neuro	23.74 (30.28)	2.71 (2.77)
21	Income/status/SES	22.81 (34.34)	2.72 (3.22)
22	Raven’s	22.80 (30.82)	2.09 (2.22)
23	Problem solving/decision making	22.76 (17.10)	3.78 (2.38)
24	Politics	22.39 (18.90)	3.23 (2.16)
25	Aggregate/regional IQs	22.04 (23.10)	2.57 (2.30)
26	ECTs (Elementary cognitive tasks)	21.95 (27.75)	1.98 (1.78)
27	Children/child development	20.11 (23.60)	2.41 (2.39)
28	SLODR (Spearman’s law of diminishing return)	19.71 (18.78)	1.95 (1.71)
29	Genes/evolution	19.65 (15.64)	2.57 (1.57)
30	Adult/aging	19.62 (22.99)	2.38 (2.54)
31	Health	19.54 (22.71)	2.17 (1.83)
32	Longitudinal designs	17.91 (19.59)	2.39 (1.90)
33	Spearman’s hypothesis	17.35 (14.89)	2.28 (1.59)
34	Nature/nurture	16.81 (18.68)	1.99 (1.88)
35	Personality	15.44 (15.54)	1.91 (1.93)
36	Race/ethnicity	13.84 (17.75)	1.81 (1.65)
37	Item level/IRT (Item response theory)	11.68 (8.73)	1.72 (1.19)

Note: “Intelligence/cognitive ability” was not included in statistical analyses for Table 2.

**Table 3:** Regression results for citations by keyword categories.

Predictor	Beta (B)	SE
Intercept	−588.4	284.8
Adult/aging	−0.20	0.40
Aggregate/regional IQs	−0.17	0.52
Attention	−0.34	0.56
Brain/neuro	−0.10	0.18
Child/Child development	−0.40	0.35
Crystallized intelligence	−0.56	0.49
ECT	−0.37	0.36
Education	0.44	0.32
Executive Function	1.85	0.44 *
Factor analysis	1.38	0.54 *
Fluid intelligence	1.22	0.54 *
Flynn effect	0.77	0.41
Genes/environment	−0.34	0.25
Genes/evolution	−0.12	0.30
g factor	−0.53	0.33
Health	−0.46	0.23 *
Income/status/SES	0.20	0.34
Intelligence/cognitive ability	0.31	0.22
IQ/achievement/aptitude test	0.20	0.27
IQ theories	1.27	0.38 *
Item level/IRT	−0.63	0.51
Longitudinal	−0.40	0.80
Memory/cognition	−0.20	0.44
Mental speed	−0.98	0.46 *
Modeling	0.51	0.37
Personality	−0.58	0.46
Politics	0.15	0.36
Psychometrics/statistics	−0.19	0.24
Race/ethnicity	−0.71	0.25 *
Raven’s	−0.71	0.56
Reasoning	−0.24	0.59
Sex differences	−0.26	0.41
Spatial ability	0.96	0.43 *
Spearman’s hypothesis	0.25	0.66
Working memory	0.93	0.34 *
Count	0.20	0.11
Year	0.29	0.14 *
Year (nonlinear)	−0.96	0.43 *
Year (nonlinear)	5.49	3.06
Year (nonlinear)	−10.52	6.78

Notes: B = Unstandardized beta; SE = standard error; model*: R*^2^ = 0.14*; R*^2^-adj = 0.10; * indicates significance at the *p* < 0.05 level. Year (nonlinear) refers to regression splines.

## Footnotes

1 In 2008, the journal’s impact factor was 3.27. As of 2017, this value was 2.79.

2 We are not criticizing authors who used redundant keywords. These are likely needed in any given study to give readers fine-grained information regarding what the article is about. These redundancies, however, complicate the coding process when the focus of the article itself (e.g., here) is on the keywords.

## Supplementary Materials

The data set is available online at https://osf.io/q8yj4/.
